# Peri-implant mucosal dehiscence coverage with a modified semilunar coronary positioned flap in posterior maxilla: a case report

**DOI:** 10.1186/s40729-015-0017-z

**Published:** 2015-06-23

**Authors:** Daisuke Ueno, Jayanetti Asiri Jayawardena, Takashi Kurokawa

**Affiliations:** 1 Department of Implantology and Periodontology, Graduate School of Dentistry, Kanagawa Dental University, 3-31-6 Tsuruya-cho, Kanagawa-ku, Yokohama Japan; 2 Department of General Education, School of Dental Medicine, Tsurumi University, Yokohama, Japan; 3 Unit of Oral and Maxillofacial Implantology, Tsurumi University Dental Hospital, Yokohama, Japan

**Keywords:** Coronary positioned flap, Connective tissue graft, Dental implant, Dehiscence coverage

## Abstract

Soft tissue dehiscence around dental implant has frequently been observed and it may lead to poor oral hygiene, especially around crowns that exhibit contours with prominent convexity. The present case demonstrates a peri-implant mucosal dehiscence coverage with modified semilunar coronary positioned flap (CPF) in #15 and 16. A semilunar partial-thickness incision was performed 7–10 mm apical from the facial gingival margin. Then, intrasulcular partial-thickness incision was tunneled to the semilunar incision. The tunnel preparation was extended interproximally under each papilla due to improvement of flap extension. Then, the tunneled flap was coronary positioned with a coronary-anchored suturing technique. Sub-epithelial connective tissue graft (SCTG) from the palate was inserted from the semilunar incision to the inside of the coronary positioned flap and sutured to stabilize the SCTG and supplemental site. Significant mucosal gain was achieved without any complication. The soft tissue volume was maintained at 9 months post-surgery, and the cleanability was improved. This technique has the potential in improving the graft survival and mucosa gain around implants.

## Introduction

Mucosal dehiscence around dental implants has frequently been observed. This phenomenon not only causes an esthetic disturbance in the anterior region but it may also lead to poor oral hygiene in the posterior region, because molar teeth exhibit contours with prominent convexities leading to accumulation of plaque. A few clinical articles on mucosal dehiscence coverage around dental implant have been published [1, 2]. Of these, only one study has been randomized controlled [3], whereas the other has been either a small a series of cases or a case report related to recession around a single implant. A literature review and a conference paper cited that dehiscence coverage around dental implant is technique-sensitive [1, 2]. Although no recommendation can be made to support the selection of one technique over another [1, 2], coronary positioned flap (CPF) with sub-epithelial connective tissue graft (SCTG) is considered to be a major treatment option. The present case report demonstrates a peri-implant mucosal dehiscence coverage technique with modified semilunar CPF in the posterior zone. This favorable result may be attributed to improved peri-operative usability and post-operative stability of the conventional technique.

## Case report

**Fig. 1:**
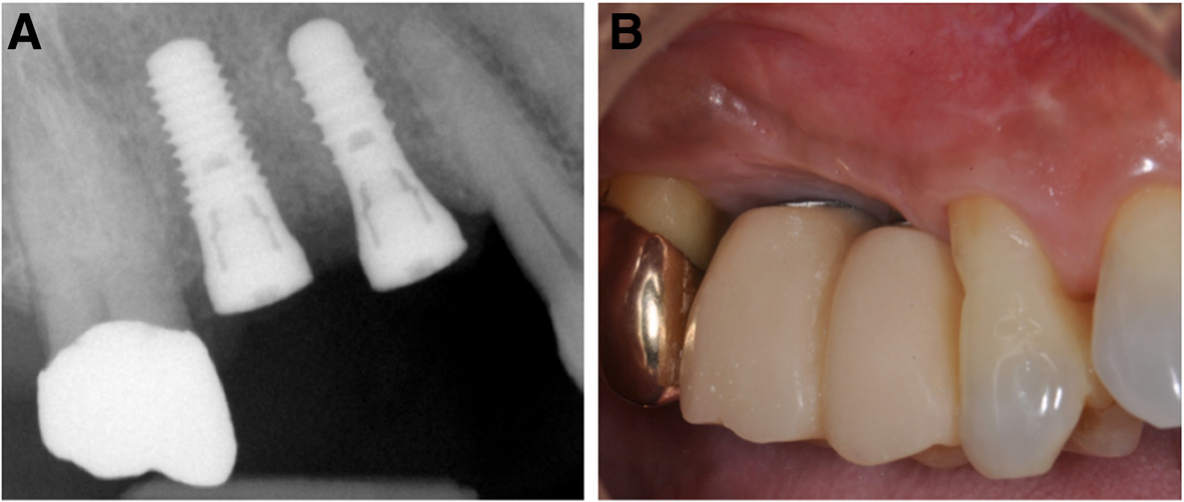
Preoperative dental X-ray (**a**) and preoperative intraoral view (**b**)

Two implants with simultaneous GBR in #15 and 16 were placed in a 65-year-old male patient in Tsurumi University Dental Hospital on September 2013 (Fig. 1a). At 5 months after implant placement, the successfully integrated implants were restored by provisional prosthesis. Since the implant-abutment connection was 1 mm supragingival and the crown contours exhibited prominent convexity, it became increasingly difficult to clean adequately around the implants (Fig. 1b). To improve the cleanability, soft tissue augmentation was carried out around the implants.

**Fig. 2:**
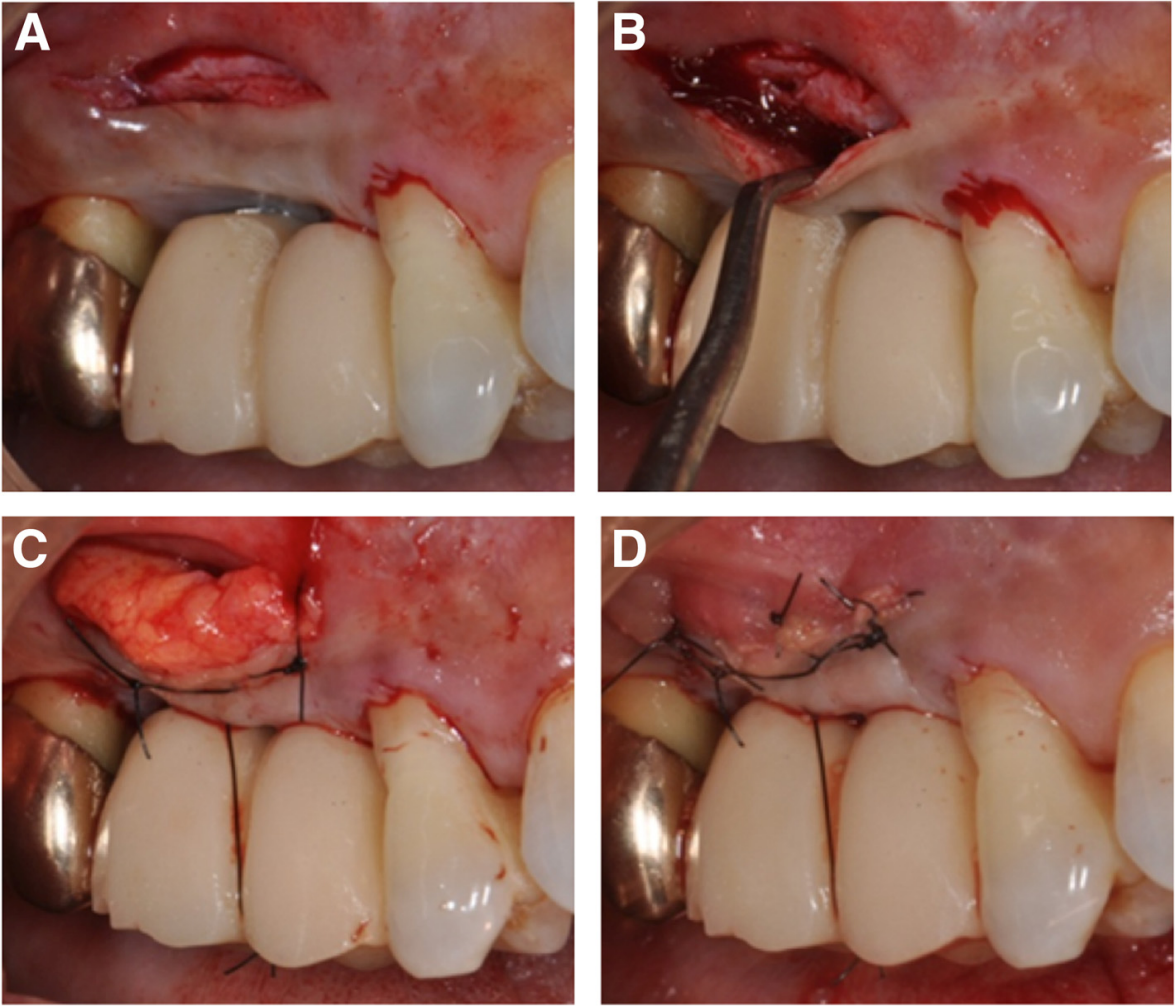
**a**–**d** Surgical steps of a modified semilunar coronary positioned flap (CPF) with sub-epithelial connective tissue graft (SCTG)

**Fig. 3:**
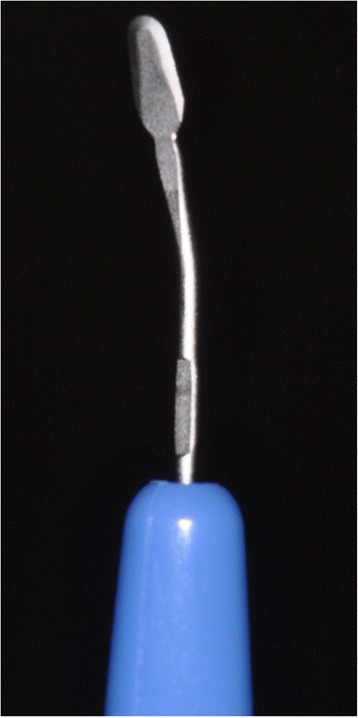
Mini-crescent knife

Following local anesthesia, a semilunar partial-thickness incision was made 7–10 mm apical from the facial gingival margin using a #15C blade (Fig. 2a). Furthermore, intrasulcular partial-thickness incision was tunneled to the semilunar incision using a mini-crescent knife^1^ (Fig. 3). The partial-thickness tunnel was extended interproximally under each papilla. After confirmation of flap extension (Fig. 2b), the 25-mm wide × 15-mm high tunneled flap was coronary positioned with a coronary-anchored suturing technique using 5.0 nylon sutures. The coronary-anchored suture was suspended and tied on a groove which was created in the coupling portion of the connecting provisional crown. A 18 × 10-mm-size SCTG was harvested from the palate in the right canine to the second molar region. The graft tissue was trimmed to fit the formerly prepared recipient bed. Then, the SCTG was inserted from the semilunar incision to the inside of the CPF (Fig. 2c) and sutured with 5.0 nylon to stabilize the SCTG and supplemental site (Fig. 2d). Suture at the access incision was removed after 2 weeks. The coronary-anchored suture was removed 2 weeks post-surgery. No complications had arisen within 3 months post-surgery.

**Fig. 4:**
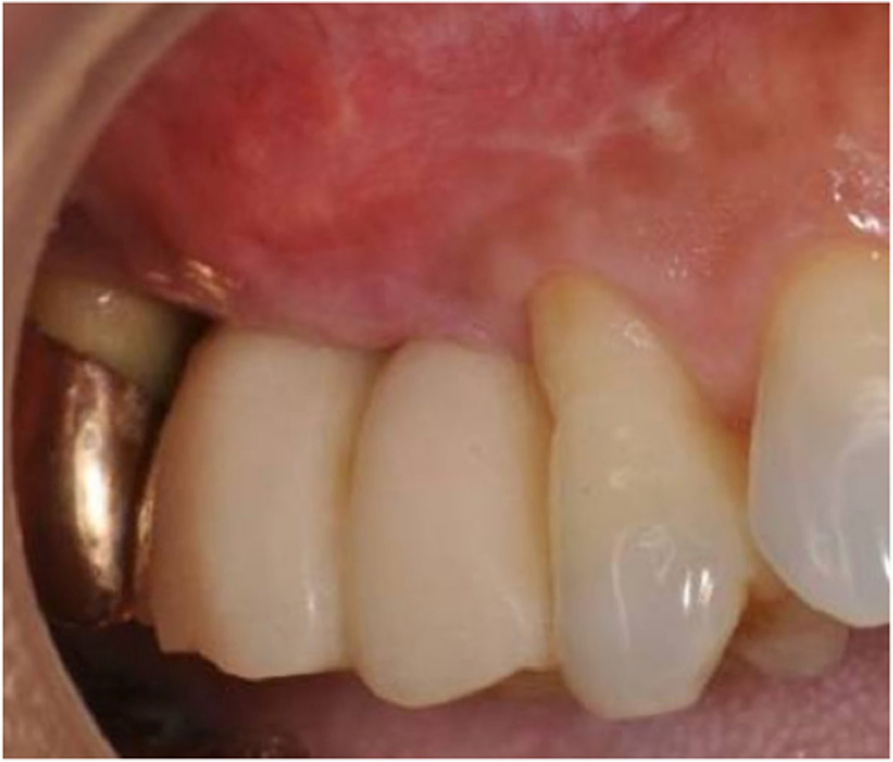
Intraoral appearance at 3 months post-surgery

**Fig. 5:**
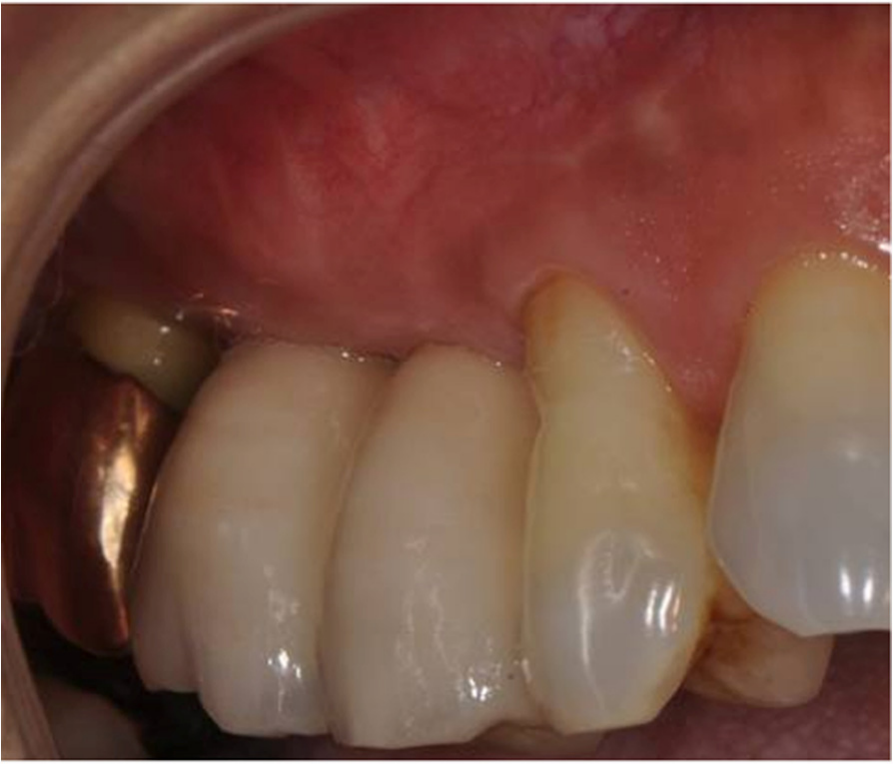
Intraoral appearance at 9 months post-surgery

Since adequate vertical and horizontal mucosal volume was gained at 3 months after the grafting (Fig. 4), the provisional prosthesis was replaced by a cement-retained metal-ceramic crown. The dehiscence sites #15 and 16 were covered successfully with regenerated mucosa. The vertical mucosal gain was 2 mm in #15 and 3 mm in #16. The horizontal mucosal gain was 2 mm in #15 and 3 mm in #16. The gain of width of keratinized mucosa was 2 mm (#15) and 2 mm (#16), respectively. At 9 months after the SCTG, the soft tissue volume was maintained, and the cleanability was improved (Fig. 5).

## Discussion

In this case, a modified semilunar CPF with SCTG successfully filled the mucogingival defect around implants. Previously, several techniques such as the rotational flap procedures (laterally sliding flap, double papilla flap, oblique rotated flap), advanced flap procedures (CPF, semilunar CPF), and regenerative procedures (with barrier membrane or application of enamel matrix proteins) have been introduced for root coverage [4]. Although all root coverage procedures can provide significant reduction in recession depth and clinical attachment (CAL) gain in Miller [5] class I and class II gingival recession, CPF in combination with SCTG is more effective than CPF in achieving root coverage and gain of keratinized mucosa [6, 7].

Only a limited number of articles have been published in peri-implant dehiscence coverage [1, 2]. Most of the cases involved autogenous mucosal graft. A conventional CPF using vertical releasing incisions (VRIs) in combination with SCTG has been considered to be a major treatment option in peri-implant dehiscence coverage. However, VRIs have risks with esthetic disturbance and post-operative course. Therefore, we previously reported a technique with coronary positioned envelope flap using SCTG [8]. The CPF with envelope technique has the advantages of increasing keratinized mucosa, a better post-operative course, and a more positive esthetic evaluation such as scars after healing than CPF with VRIs [9]. In contrast, coronary positioning of flap is difficult in cases with scarring recipient mucosa and multi-implant recession due to less flap mobility. In such cases, additional semilunar incision is useful in improving the flap mobility. Coronary-anchored suture (CAS) facilitates to make space for insertion of connective tissue and to fix the position of CPF. Since reduction of micromotion during healing period influences graft survival, CAS may be useful in envelope and tunneling techniques. Further studies are needed to evaluate the usefulness of this technique.

## Footnotes

1 Mini-crescent angled knife (size 1.25 mm), Sharpoint™, PA, USA
